# Case Report: Low-dose mitotane in a case of recurrent adrenocortical carcinoma: a 20-year clinical perspective

**DOI:** 10.3389/fonc.2026.1826235

**Published:** 2026-08-11

**Authors:** Tomasz Buczek, Ewa Kilar-Kobierzycka, Jowita Halupczok-Żyła, Marek Bolanowski

**Affiliations:** 1 Department of Clinical Pharmacology, Wroclaw Medical University, Wroclaw, Poland; 2 Department of Clinical and Experimental Pathology, Wroclaw Medical University, Wroclaw, Poland; 3 Department and Clinic of Endocrinology and Internal Medicine, Wroclaw Medical University, Wroclaw, Poland

**Keywords:** adrenal insufficiency, adrenocortical carcinoma, long-term treatment, mitotane, monitored therapy

## Abstract

Adrenocortical carcinoma (ACC) is a rare malignancy with a high risk of recurrence, and the prolonged use of mitotane is often limited by its narrow therapeutic window and endocrine and gastrointestinal toxicity. We describe a woman who presented at 42 years of age with a right adrenal mass in 2000. The tumour was macroscopically removed in 2001 and was diagnosed as a 7.4 × 5.6 × 6.0 cm, partially necrotic ACC with focal calcifications, invasion of the surrounding adipose tissue, and microscopically involved margins (pT3N0M0). A local recurrence was resected in 2006, after which mitotane was initiated at 6 g/day. Gastrointestinal intolerance required reduction to 3 g/day and subsequently to 1–2 g/day, with a temporary interruption in 2009. A contralateral adrenal lesion was removed in 2011 and proved to be an adenoma, resulting in bilateral adrenalectomy and lifelong glucocorticoid and mineralocorticoid replacement. Mitotane concentrations remained subtherapeutic for many years but reached 16.9 mg/L in 2021 and 20.3 mg/L in 2022 on 2 g/day. Mitotane was discontinued on 5 June 2025 after approximately 19 years of treatment. At the latest assessment, the patient remained in good general condition and continued hydrocortisone 40 mg/day, fludrocortisone 0.1 mg/day, and levothyroxine. Contrast-enhanced computed tomography on 28 May 2026 showed no local recurrence or metastatic disease. This case documents sustained disease control during and after almost 20 years of individualized mitotane therapy following recurrent ACC. It emphasizes the importance of dose adaptation, therapeutic drug monitoring, careful endocrine replacement, and cautious interpretation of long-term benefit in a single patient.

## Introduction

Adrenal masses are increasingly detected with cross-sectional imaging; their prevalence is approximately 1-4% in routinely imaged adults and rises substantially with age. Most are benign adenomas, whereas adrenocortical carcinoma (ACC) accounts for only a small proportion of adrenal lesions (1). ACC itself has an estimated annual incidence of approximately 0.7–2 cases per million people, occurs most often in the fourth to fifth decades of life, and is reported more frequently in women than in men (2, 3).

Complete surgical resection is the principal potentially curative treatment. Nevertheless, recurrence is common, particularly after locally advanced disease, tumour spillage, high proliferative activity, or incomplete resection. Mitotane is used as adjuvant therapy in patients at increased risk of recurrence and as systemic treatment for recurrent or advanced disease, but its highly variable pharmacokinetics, long half-life, narrow therapeutic range, drug interactions, and frequent adverse effects complicate long-term administration (4–7).

Reports describing nearly two decades of continuous mitotane exposure after a documented ACC recurrence are uncommon. We present a 20-year treatment course characterized by early intolerance, prolonged use of low daily doses, delayed achievement of target plasma concentrations, bilateral adrenalectomy, complex hormone replacement, and sustained radiological disease control.

## Case description

### Initial presentation and primary tumour

In 2000, a 42-year-old woman was evaluated for episodes of severe arterial hypertension, with blood pressure up to 200/120 mmHg, palpitations, excessive sweating, and facial flushing. Abdominal ultrasonography on 11 August 2000 showed a heterogeneous, smoothly encapsulated mass measuring 66 × 46 mm above the upper pole of the right kidney, corresponding to the right adrenal gland; no other abdominal lesions were identified. A 24-hour urinary vanillylmandelic acid concentration was within the reference range at 5.34 mg/24 h. The surviving records did not contain results of plasma or urinary metanephrines, a dexamethasone suppression test, or a complete assessment of cortisol, aldosterone, renin, and adrenal androgens at initial diagnosis. The tumour was therefore described in the archival documentation as clinically non-functioning, although its hormonal status cannot now be fully reconstructed. The presenting symptoms were treated as non-specific findings that prompted adrenal evaluation rather than as evidence of a confirmed secretory syndrome.

Right adrenal surgery was performed on 2 April 2001. The operative record described macroscopically complete removal of the lesion. Archival histopathological documentation identified an adrenocortical carcinoma measuring 7.4 × 5.6 × 6.0 cm, partially necrotic and containing focal calcifications. The carcinoma invaded the surrounding adipose tissue and was not removed within histologically tumour-free margins. The tumour was classified as pT3N0M0. No postoperative systemic therapy was initiated; the rationale was not recorded in the surviving documentation. The patient remained under endocrine follow-up between 2001 and 2006, but detailed records from this interval were unavailable.

The patient reported no family history of ACC, adrenal tumours, or other malignancies. Genetic testing was not performed because the clinical and family history available at the time did not raise suspicion of a hereditary cancer syndrome.

### Local recurrence and initiation of mitotane

A local recurrence in the right adrenal bed was identified in early 2006. On 6 February 2006, the recurrent tumour was resected with completion right adrenalectomy. The available histopathological summary reported carcinomatous infiltration consistent with recurrent ACC. The full report and tissue material were not available for retrospective comparison with the primary tumour; consequently, the mitotic count, Ki-67 index, Weiss score, and margin status could not be reassessed.

Postoperative endocrine testing on 22 March 2006 showed testosterone 0.2 ng/mL, dehydroepiandrosterone sulfate (DHEA-S) 21.2 µg/dL, adrenocorticotropic hormone (ACTH) 13.0 pg/mL, supine aldosterone 110.3 pg/mL, upright aldosterone 160.7 pg/mL, and urinary aldosterone 10.86 µg/24 h. Mitotane was initiated in April 2006 at 6 g/day using a high-dose approach. Hydrocortisone 20 mg each morning was introduced concurrently. Gastrointestinal toxicity, including nausea, vomiting, and diarrhoea, necessitated reduction of mitotane to 3 g/day by November 2006 and an increase in hydrocortisone to 30 mg/day. On 9 November 2006, ACTH was 317 pg/mL and DHEA-S remained low, supporting insufficient glucocorticoid replacement despite measurable serum cortisol during hydrocortisone therapy.

In May 2007, ultrasonography showed no pathological lesion in either adrenal region or in the postoperative right adrenal bed. Mitotane was reduced to 2 g/day by March 2008. In March 2009, treatment was temporarily discontinued because of dizziness, nausea, and vomiting. Fluorodeoxyglucose positron-emission tomography showed no active neoplastic disease. Mitotane was restarted in July 2009 at 1.5 g/day, together with hydrocortisone 30 mg/day.

### Contralateral adrenal lesion and endocrine replacement

In March 2011, computed tomography detected a left adrenal lesion measuring approximately 3.4 cm; ultrasonography showed a 2.7 × 1.8 cm lesion, and a repeat examination on 10 May 2011 measured it at 2.0 × 2.5 cm. The patient reported malaise, nausea, chest pain, and headache. Hormonal evaluation did not establish a new secretory syndrome. A 24-hour urinary free cortisol concentration was elevated at 241.1 ng/24 h, but the test was performed during hydrocortisone replacement and was therefore not interpretable as endogenous cortisol production. Plasma catecholamines were within the available reference ranges. Because of the history of recurrent ACC and the uncertain nature of a new contralateral adrenal mass, left adrenalectomy was performed on 12 May 2011. The available documentation recorded a final diagnosis of adrenal adenoma. The complete histopathological report was unavailable for retrospective review.

Bilateral adrenalectomy required lifelong glucocorticoid and mineralocorticoid replacement. In July 2011, ACTH remained markedly elevated at 553 pg/mL despite hydrocortisone 30 mg/day, and dexamethasone 0.25 mg every other morning was briefly added. It was subsequently stopped, and fludrocortisone 0.05 mg every other day was introduced because of elevated plasma renin activity. When ACTH increased to 691 pg/mL in December 2011, low-dose dexamethasone 0.25 mg at bedtime twice weekly was added to hydrocortisone and fludrocortisone. Dexamethasone was later withdrawn because of insulin resistance and metabolic complications. Hydrocortisone was increased to as much as 50 mg/day, and fludrocortisone was adjusted according to clinical status, electrolytes, and renin.

### Long-term treatment, follow-up, and outcomes

Magnetic resonance imaging in August 2012 and computed tomography in June 2014 showed no recurrence. Mitotane was continued at 1-1.5 g/day despite plasma concentrations of 3.5 mg/L in 2013 and 3.4 mg/L in 2014. The dose was increased to 2 g/day in 2016; the plasma concentration was 9.1 mg/L in February 2016. During this period, weakness, nausea, anorexia, and weight loss were attributed partly to inadequate glucocorticoid replacement rather than mitotane toxicity alone.

The patient experienced recurrent episodes of adrenal decompensation and electrolyte disturbance. In August 2018, she was hospitalized with profound weakness that prevented walking and a serum sodium concentration of 129.6 mmol/L while receiving hydrocortisone 30 mg/day. Treatment included fluid and electrolyte correction and adjustment of replacement therapy. Relevant comorbidities during follow-up included type 2 diabetes mellitus, metabolic syndrome, hypertension, hypothyroidism, osteoporosis, and depression.

Mitotane 2 g/day was maintained from 2016 until 5 June 2025, when treatment was discontinued after approximately 19 years of cumulative therapy. The first documented concentration within the usual target range was 16.9 mg/L in June 2021; it was 20.3 mg/L in June 2022. At the most recent assessment in 2026, the patient was in good general condition and continued hydrocortisone 40 mg/day, fludrocortisone 0.1 mg each morning, and levothyroxine 75 µg each morning from Monday to Friday. Serum sodium and potassium were within their reference ranges. Contrast-enhanced helical computed tomography of the chest, abdomen, and pelvis on 28 May 2026, approximately one year after mitotane discontinuation, showed no recurrence in either adrenal bed, no lymphadenopathy, and no distant metastatic disease. The major clinical events, selected hormonal and electrolyte findings, treatment modifications, and follow-up outcomes are summarized in **Table 1**. Mitotane dose adjustments and available plasma concentrations are presented in **Table 2**.

**Table 1:** Clinical timeline, selected biochemical findings, treatment, and follow-up.

Date	Clinical event	Clinical, biochemical, and management details
August 2000	Symptoms and initial imaging	Severe episodic hypertension, palpitations, excessive sweating, and facial flushing. US showed a heterogeneous right adrenal mass measuring 66 × 46 mm, with no other abdominal lesions. Urinary VMA was 5.34 mg/24 h and within the reference range. Results of DST and plasma or urinary metanephrine testing were unavailable.
April 2001	Primary surgery	Macroscopically complete right adrenal tumour removal. Histology: ACC 7.4 × 5.6 × 6.0 cm, partial necrosis, focal calcifications, invasion of surrounding fat, involved microscopic margins; pT3N0M0.
2001-2005	Surveillance	Endocrine follow-up documented, but detailed clinical, hormonal, and imaging records were unavailable.
February-April 2006	Local recurrence and systemic treatment	Resection with completion right adrenalectomy was performed on 6 February. The available histopathological summary documented carcinomatous infiltration consistent with recurrent ACC. On 22 March, testosterone was 0.2 ng/mL, DHEA-S 21.2 µg/dL, ACTH 13.0 pg/mL, supine aldosterone 110.3 pg/mL, upright aldosterone 160.7 pg/mL, and urinary aldosterone 10.86 µg/24 h. Mitotane was initiated in April at 6 g/day together with hydrocortisone replacement.
May-November 2006	Early endocrine monitoring and dose reduction	On 15 May, ACTH was 36.7 pg/mL and DHEA-S 15 µg/dL. Serum cortisol was 5.25 µg/dL at 05:30, 9.34 µg/dL at 08:00, 4.82 µg/dL at 20:00, and 3.11 µg/dL at 24:00. PRA was 3.37 ng/mL/h supine and 3.90 ng/mL/h upright. On 9 November, ACTH increased to 317 pg/mL; serum cortisol was 14.17 µg/dL at 05:30, 14.04 µg/dL at 08:00, 11.19 µg/dL at 20:00, and 3.96 µg/dL at 24:00. Cortisol was measured during hydrocortisone replacement and after the morning dose. Mitotane was reduced to 3 g/day because of vomiting and diarrhoea, and hydrocortisone was increased to 30 mg/day.
May 2007-March 2008	Dose reduction	In May 2007, ACTH was 12.3 pg/mL and DHEA-S 15 µg/dL; US showed no recurrent lesion. In March 2008, PRA was 0.55 ng/mL/h supine and 4.15 ng/mL/h upright, while aldosterone was 9.7 pg/mL supine and 31.74 pg/mL upright. Mitotane was reduced to 2 g/day.
March-July 2009	Temporary interruption	Mitotane stopped because of dizziness, nausea, and vomiting. FDG-PET negative. Treatment restarted at 1.5 g/day.
September 2010	Endocrine follow-up	During treatment with mitotane 1.5 g/day and hydrocortisone 30 mg/day, serum cortisol was 5.44 µg/dL at 06:00, 9.41 µg/dL at 08:00, 3.41 µg/dL at 20:00, and 1.32 µg/dL at 24:00. Samples were obtained after hydrocortisone administration.
2011	Contralateral adrenal lesion	A left adrenal lesion measuring approximately 3.4 cm was detected on CT. Before surgery, urinary free cortisol was 241.1 ng/24 h, PRA was 0.49 ng/mL/h supine and 0.79 ng/mL/h upright, and aldosterone was 9.2 pg/mL supine and 14 pg/mL upright. Left adrenalectomy was performed on 12 May; the available histopathological documentation identified an adrenal adenoma. Following bilateral adrenalectomy, ACTH was 553 pg/mL in July and 691 pg/mL in December. Hydrocortisone, temporary low-dose dexamethasone, and fludrocortisone were adjusted.
2012-2014	Continued low-dose therapy	In July 2012, ACTH was 75 pg/mL and DHEA-S <15 µg/dL. MRI in 2012 and CT in 2014 showed no recurrence. Mitotane was continued at 1-1.5 g/day; plasma concentrations were 3.5 mg/L in 2013 and 3.4 mg/L in 2014.
2016	Dose escalation	Mitotane was increased to 2 g/day; its plasma concentration was 9.1 mg/L. Sodium was 135 mmol/L, potassium 4.8 mmol/L, ACTH 1004 pg/mL, and PRA 15.26 ng/mL/h supine and 68.38 ng/mL/h upright. Hydrocortisone replacement was increased because of symptoms and biochemical findings suggesting insufficient replacement.
August 2018	Adrenal decompensation	Hospitalization for profound weakness and hyponatremia (sodium 129.6 mmol/L). Replacement and electrolytes adjusted.
2021-2022	Target mitotane concentrations	Mitotane was maintained at 2 g/day. Plasma concentrations were 16.9 mg/L in June 2021 and 20.3 mg/L in June 2022. In June 2022, sodium was 137 mmol/L, potassium 3.2 mmol/L, direct renin 500 µIU/mL, and aldosterone 0.97 ng/dL. Hydrocortisone and fludrocortisone replacement were adjusted individually.
June 2025	Biochemical follow-up and mitotane discontinuation	Sodium was 137 mmol/L, potassium 4.0 mmol/L, serum cortisol 0.3 µg/dL, direct renin 500 µIU/mL, testosterone 0.2 ng/mL, and DHEA-S 16 µg/dL. The timing of cortisol sampling relative to the hydrocortisone dose was not documented. Mitotane was discontinued on 5 June 2025 after approximately 19 years of treatment.
May 2026	Latest follow-up	The patient was in good general condition and continued hydrocortisone 40 mg/day, fludrocortisone 0.1 mg each morning, and levothyroxine 75 µg each morning from Monday to Friday. Serum sodium and potassium were within the reference ranges. CT of the chest, abdomen, and pelvis, performed approximately one year after mitotane discontinuation, showed no local recurrence, lymphadenopathy, or distant metastatic disease.

ACC, adrenocortical carcinoma; ACTH, adrenocorticotropic hormone; CT, computed tomography; DHEA-S, dehydroepiandrosterone sulfate; DST, dexamethasone suppression test; FDG-PET, 18F-fluorodeoxyglucose positron-emission tomography; MRI, magnetic resonance imaging; PRA, plasma renin activity; US, ultrasonography; VMA, vanillylmandelic acid. Serum cortisol concentrations obtained in 2006 and 2010 were measured during hydrocortisone replacement and after the morning dose and therefore do not represent endogenous adrenal cortisol secretion. Urinary free cortisol in 2011 was also measured during hydrocortisone replacement. Reference ranges: serum cortisol before 10:00, 3.7-19.4 µg/dL; after 17:00, 2.9-17.3 µg/dL; urinary free cortisol, 13.7-75.3 ng/24 h; PRA supine, 0.5-1.9 ng/mL/h; PRA upright, 1.9-6.0 ng/mL/h; aldosterone supine, 10–105 pg/mL; aldosterone upright, 34–273 pg/mL.

**Table 2:** Mitotane dose, plasma concentration, and treatment modifications.

Date	Dose (g/day)	Plasma level (mg/L)	Notes
April 2006	6.0	Not available	Treatment initiated after resection of local recurrence.
November 2006	3.0	Not available	Dose reduced because of vomiting and diarrhoea.
March 2008	2.0	Not available	Further dose reduction for tolerability.
March 2009	0	–	Temporarily discontinued because of dizziness, nausea, and vomiting.
July 2009	1.5	Not available	Reintroduced after FDG-PET excluded active disease.
2013	1.0	3.5	Subtherapeutic concentration.
June 2014	1.5	3.4	Persistently subtherapeutic concentration; CT showed no recurrence.
February 2016	2.0	9.1	Dose maintained despite concentration below target because of prior intolerance.
January 2020	2.0	Not available	Stable long-term dose.
June 2021	2.0	16.9	Target concentration achieved.
June 2022	2.0	20.3	Slightly above the usual target range.
June 2024	2.0	Not available	Dose maintained.
May 2026	0	Not available	Mitotane discontinued after approximately 19 years of treatment.

The generally recommended target plasma concentration is 14–20 mg/L. FDG-PET, 18F-fluorodeoxyglucose positron-emission tomography; CT, computed tomography.

## Discussion

This case is notable for sustained disease control after a documented local recurrence and almost 20 years of mitotane exposure, much of it at daily doses of 1–2 g and at measured concentrations below the conventional target. The course also illustrates the practical difficulty of separating mitotane toxicity from manifestations of adrenal insufficiency, particularly nausea, anorexia, weakness, dizziness, and electrolyte abnormalities.

Mitotane is a dichlorodiphenyltrichloroethane (DDT) derivative with selective adrenolytic and steroidogenesis-inhibiting effects. Its complete mechanism remains incompletely defined. Experimental data indicate disruption of mitochondrial function and cholesterol homeostasis, inhibition of steroidogenic pathways, and inhibition of sterol-O-acyl transferase 1, leading to free-cholesterol accumulation, endoplasmic-reticulum stress, and apoptosis in adrenocortical cells (8, 9). Mitotane also strongly induces CYP3A4 and alters steroid metabolism, which accelerates hydrocortisone inactivation and contributes to clinically important drug interactions (4, 10).

Therapeutic drug monitoring is central to treatment because mitotane absorption and accumulation vary widely. Current guidelines generally aim for a trough concentration of 14–20 mg/L, with measurement every 3–4 weeks during dose escalation and every 2–3 months after a plateau has been reached; neurological toxicity becomes more common above 20 mg/L (4, 5, 11, 12). Low-dose escalation can achieve target concentrations with improved tolerability in some patients (13). In the present case, the target range was not documented until 2021, 15 years after treatment initiation. The absence of recurrence during prolonged subtherapeutic exposure does not prove that low plasma concentrations were sufficient. Surgical control of the recurrence, favourable tumour biology, incomplete sampling of historical concentrations, and the cumulative tissue burden of a highly lipophilic drug may all have contributed. Rare responses at concentrations below 14 mg/L have been reported, but they remain unpredictable and should not be interpreted as a reason to abandon standard monitoring targets (14).

The optimal duration of mitotane is uncertain. Guidelines generally suggest 2–5 years of adjuvant treatment after high-risk resection when tolerated, whereas duration in recurrent disease is individualized and may be substantially longer (4, 5). This patient’s prior recurrence, the microscopically involved margin after the primary surgery, absence of alternative systemic therapy, and acceptable later tolerability supported prolonged treatment. Mitotane was ultimately discontinued on 5 June 2025 after approximately 19 years of therapy. Such a duration substantially exceeds conventional adjuvant practice and illustrates the importance of regular reassessment of the individual risk-benefit balance.

Endocrine replacement was a major component of care. Guidelines recommend glucocorticoid replacement for nearly all patients receiving mitotane and note that at least twice the usual replacement dose is often required because of increased steroid clearance and increased cortisol-binding globulin (4, 10). Serum cortisol is difficult to interpret during hydrocortisone therapy and mitotane exposure; dosing should therefore be guided predominantly by clinical assessment, with ACTH used as supportive information. Mineralocorticoid replacement is guided by symptoms, blood pressure, sodium, potassium, and renin. Thyroid function should also be monitored because mitotane can cause central hypothyroidism and alter thyroid hormone-binding proteins (4). In this patient, persistently elevated ACTH led to brief use of low-dose dexamethasone in addition to hydrocortisone. It was discontinued because of insulin resistance and metabolic complications. Recurrent hyponatremia, including the 2018 hospitalization, underscored the need for repeated adjustment of hydrocortisone and fludrocortisone.

Long survival despite recurrent ACC has been described. Hermsen et al. reported six patients living 12–28 years after recurrent or metastatic disease, often after repeated surgery and mitotane (15). Wängberg et al. also emphasized active surgical management and monitored mitotane in long-term survivors (16). More recent institutional data confirm that resection status, tumour stage, proliferative activity, and achievement of therapeutic mitotane concentrations are major prognostic factors (17). At the same time, mitotane treatment is associated with substantial and heterogeneous toxicity, frequently requiring dose modification and close clinical and biochemical monitoring (18). Despite advances in multidisciplinary management, systemic treatment options for recurrent or advanced ACC remain limited, and therapeutic decisions must therefore be individualized (19). Our case adds a detailed endocrine and pharmacological perspective, including bilateral adrenalectomy, prolonged low-dose exposure, delayed therapeutic concentrations, and long-term management of replacement-related complications.

The principal strength of this report is the duration of observation, supported by serial treatment records, mitotane concentrations, endocrine data, and current cross-sectional imaging. Important limitations are its retrospective nature; missing complete histopathological reports from 2006 and 2011; absence of Ki-67 and a formal retrospective pathological review; incomplete hormonal testing and unavailable CT attenuation and washout data at initial diagnosis; assay changes over time; and lack of germline testing. Current guidance recommends at least a basic clinical genetic evaluation in adults with ACC, although this patient had no suggestive personal or family history (4). Finally, a single case cannot establish that prolonged low-dose mitotane caused the favourable outcome.

## Patient perspective

The patient provided the following perspective: “Living with recurrent adrenocortical carcinoma and long-term mitotane therapy has been challenging. At the beginning, gastrointestinal adverse effects made daily life difficult and required several dose changes. Lifelong adrenal insufficiency and electrolyte disturbances have required constant attention and occasional hospital treatment. Despite these burdens, I am reassured that the cancer has not returned for many years. Regular monitoring and support from the multidisciplinary team helped me remain on treatment for many years and manage its complications. I hope that sharing my experience will help other patients understand the importance of adherence, monitoring, and asking for help when symptoms change.”

## Conclusion

Long-term disease control after recurrent ACC can coexist with substantial treatment toxicity and complex endocrine replacement needs. This nearly 20-year mitotane course, completed in June 2025, supports an individualized approach based on tolerability, serial plasma concentrations, radiological surveillance, and active management of glucocorticoid, mineralocorticoid, thyroid, and electrolyte disturbances. The favourable outcome should be interpreted as an association within a single case rather than evidence that subtherapeutic mitotane concentrations are generally adequate.

## Data Availability

The raw data supporting the conclusions of this article will be made available by the authors, without undue reservation.
